# Double-row wired anchors combined with “8” cross tension bands: an innovative procedure for the treatment of inferior pole fractures of the patella and its finite element and biomechanical evaluation

**DOI:** 10.1007/s00402-026-06283-7

**Published:** 2026-04-20

**Authors:** Zihao Deng, Wei Zhang, Jian Chen, Zilong Yang, Wentao Gao, Yuxuan Liu, Xiping Zhang, Wei Zeng, Zhiqin Liu

**Affiliations:** 1 Trauma Center Department Three, Zhuzhou Hospital Affiliated to Xiangya School of Medicin, Central South University, Zhuzhou, Hunan China; 2 Trauma Center Department Four, Zhuzhou Hospital Affiliated to Xiangya School of Medicine, Central South University, Zhuzhou, Hunan China; 3 People’s Hospital of Lukou District, Zhuzhou, Hunan China

**Keywords:** Patellar fracture, Inferior pole, Anchor, Finite element analysis, Mechanical analysis

## Abstract

**Objective:**

To propose and verify the biomechanical properties of a new double-row suture anchor combined with figure-of-eight cross tension band technology for the treatment of inferior pole fractures of the patella, in order to overcome the shortcomings of traditional fixation methods in achieving stable fixation and reducing implant-related complications.

**Methods:**

(1) Based on clinical CT data, a three-dimensional finite element model of patellar inferior pole fracture was reconstructed, and the new fixation technology (deep double-row anchors in the proximal bone fragment combined with figure-of-8 sutures) and Kirschner wire tension band wire fixation (TBW) were simulated and analyzed. Mechanical performance in the treatment of inferior pole fracture of the patella; (2) Divide the model into two groups according to surgical methods: suture anchor (SA) fixation group and TBW fixation group; (3) Carry out finite element analysis to compare the two groups at 500 Displacement and stress distribution of the knee joint at 45°and 135°of flexion under N load.

**Results:**

Finite element analysis and biomechanical test results showed that there was no significant difference in displacement and stress between the two groups at different flexion angles.

**Conclusion:**

Double-row anchors combined with figure-of-eight suture technology exhibited excellent biomechanical properties, and is a feasible fixation solution for the treatment of inferior pole fractures of the patella.This technology can significantly reduce stress concentration and the risk of implant complications, providing a minimally invasive solution to replace traditional metal implants and helping patients recover early, but its long-term efficacy still requires further clinical verification.

## Introduction

As an important fulcrum for knee flexion and extension activities, the patella plays a key role in the knee extensor system [1]. Therefore, destroying the integrity of the patella or interfering with its normal movement trajectory can significantly damage knee joint function, leading to chronic pain and even more serious complications [2]. Among them, inferior patellar fracture is a special type of patellar fracture, which is classified as an extra-articular fracture because the fracture line is usually located outside the joint capsule, accounting for approximately 9.3–22.4% of all patellar fracture cases [3]. The distinctive feature of this type of fracture is that the fracture fragments are mostly comminuted fragments, which makes it difficult to achieve effective anatomical reduction and stable fixation with conservative treatment [4]. Therefore, early surgical intervention is widely considered to be the preferred option in clinical practice.

However, the unique anatomical structure of the inferior pole of the patella and the comminuted nature of the fracture fragment pose great challenges to achieving strong internal fixation [5]. Currently commonly used surgical methods include TBW and lower pole resection [6]. However, because lower pole resection will change the normal anatomical structure of the patella and easily lead to problems such as patellar instability, its application has been significantly reduced [7]. At the same time, for lower-level fractures with severe comminution, traditional TBW technology is often difficult to obtain reliable fixation results because the fracture fragments are too small and too fragmented. Therefore, there is no broad consensus on the optimal treatment of inferior patellar fractures and a lack of unified standards. In recent years, a variety of novel fixation techniques have been introduced into clinical practice to address the challenges of comminuted fractures. For example, for severe comminuted fractures where TBW often fails, Hu et al. reported that the application of "candy box" technology achieved biomechanical stability that is equivalent to or even better than tension band fixation [8]. Gao and his team used Kirschner wires combined with modified tension band technology to treat comminuted fractures of the inferior patella, and also reported good clinical efficacy [9]. In addition, suture anchor fixation technology exhibits significant advantages in terms of fixation strength. Research by Zhang et al. has shown that the application of suture anchor fixation can achieve a more reliable and stable fixation effect [10]. Therefore, suture anchor technology is gaining increasing attention as a versatile option with the advantage of better preserving patellar anatomy while reducing the risk of complications such as implant protrusion. Furthermore, this approach not only addresses the mechanical challenges associated with traditional fixation methods, but also aligns with contemporary trends in orthopedic surgery that emphasize minimally invasive techniques and enhanced functional outcomes.

Today, the development of computed tomography technology and advanced medical imaging software has revolutionized fracture analysis methods. This technology enables precise recording of the size, shape, number, and spatial orientation of fracture fragments, thereby providing valuable morphological insights into fracture morphological characteristics. Fracture mapping has become an important tool in orthopedic surgery, helping surgeons more accurately understand fracture patterns and guide the selection of the most appropriate and effective surgical plan. Although this technology is increasingly used in the treatment of various types of fractures, there are currently no reports in the literature on the systematic application of fracture mapping technology in this type of fractures. Given its complex structure and often comminuted nature, the application of fracture mapping can significantly enhance preoperative planning and improve surgical outcomes by enabling more individualized fixation protocols. This study aims to systematically apply fracture mapping technology to this type of fracture to fill the research gap in this field and provide a basis for more personalized and efficient treatment strategies [11].

Based on this, this study uses finite element analysis and biomechanical experiments to compare the new internal fixation method with the traditional TBW technology under the same boundary conditions, and systematically evaluates the displacement of the fracture fragment and the stress distribution of the internal fixation, thereby identifying a more reliable internal fixation method for patellar inferior pole fractures.

## Method

### Data acquisition and 3D reconstruction

**Fig. 1:**
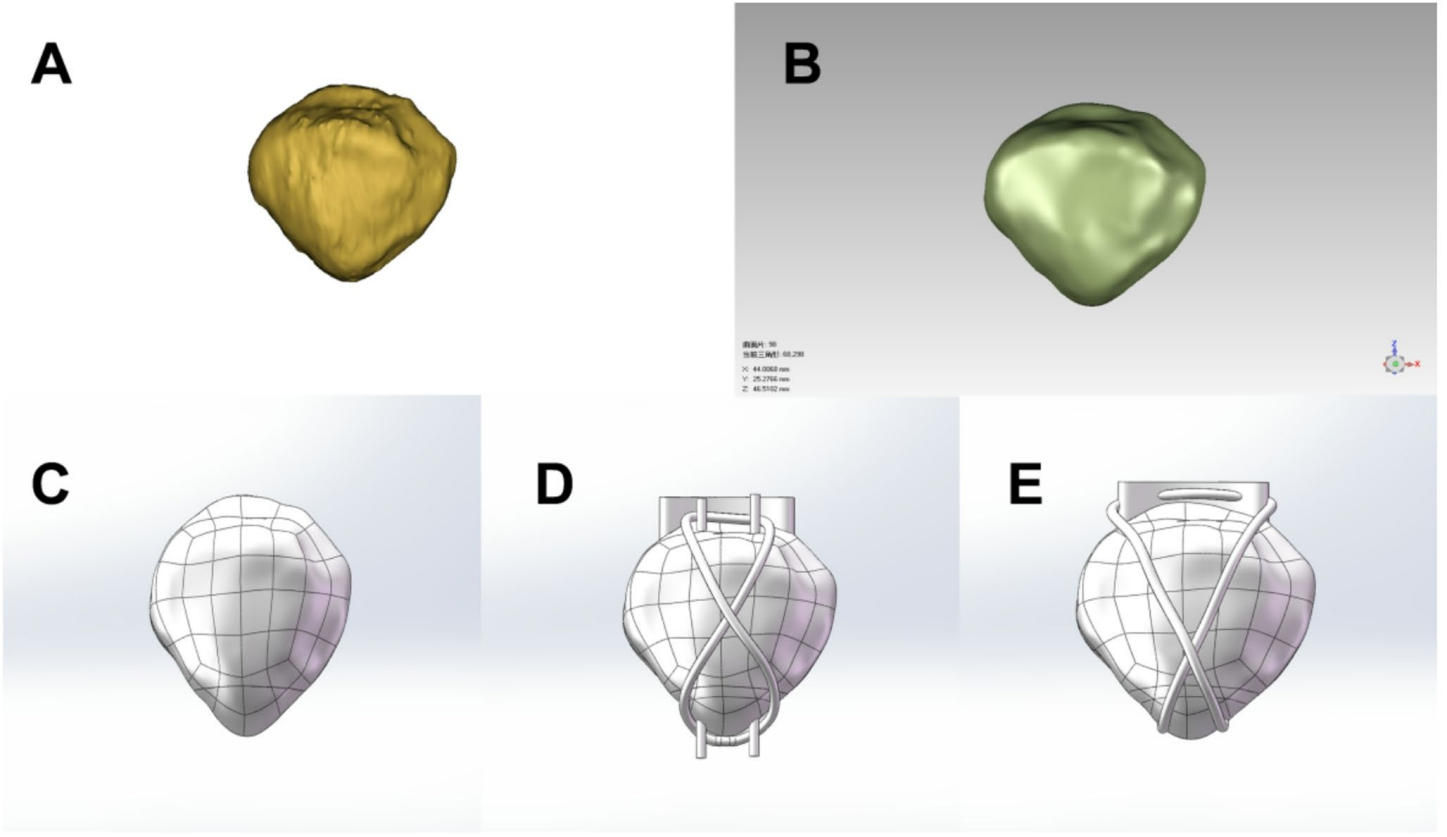
Reconstructed 3D fracture model. (**A**) 3D image of patella, (**B**) Relaxation and surface processing, (**C**–**E**) Construction of normal patella, TBW and SA models

This study is based on knee CT image data of a healthy young male volunteer with no history of knee pain or trauma. A 128-slice spiral computed tomography scanner (SIMENS SENSATION 128, slice thickness: 0.6 mm, slice gap: 0.6 mm, resolution: 512*512 pixels) was used to perform thin-slice scans from thigh to calf on supine adult male volunteers with the knee joint in a neutral position. The obtained two-dimensional image data were stored in DICOM format and imported into the 3D image modeling software processing system Mimics21 (Fig. 1A). The patellar area was selected through region growing, the patellar fracture line was extracted, and a 3D model of the patella and patellar inferior pole fracture was established. The model was then imported into Geomagic Studio 2021 software for noise removal, packaging, and smoothing, and the software's surface modeling capabilities were used to create volumetric mesh entities (Fig. 1B).

### Model construction

The stp file obtained in Geomagic Wrap 2021 was imported into Solidworks 2024 (Dassault Systèmes, USA), and the cortical bone and cancellous bone were assembled to obtain a complete patella model (Fig. 1C). After that, the TBW and SA models were established in Solidworks respectively. Among them, the TBW model: two 2.0 mm diameter Kirschner wires are placed parallel to the articular surface of the patella, and then two 2.0 mm diameter steel cables are wound around the patella in a figure-8 shape to construct a tension band fixation system to achieve stable compression of the fracture fragment; the SA model: use two 5 mm anchors, which are symmetrically placed proximal to the patellar fracture line. Two sutures are attached to each anchor, and a bone tunnel is then prepared under the anchor so that the sutures pass through the tunnel and run along the inferior pole of the patella. The sutures were crossed diagonally to the opposite side, paired and knotted sequentially to form an "8" fixed structure, and finally the ligation was reinforced again (Fig. 1D, E). Finally, the obtained geometric model is saved in XT format and imported into ANSYS to set material properties, contact relationships, remeshing, applied forces and boundary conditions for finite element calculation. The material properties refer to relevant literature data [12]. Material properties are shown in Table 1.

**Table 1:** Model material parameters

Name of the material	Elastic modulus (MPa)	Poisson ratio
Cortical bone	10,000	0.3
Cancellous bone	840	0.29
Cartilage	5	0.45
Muscle	215.3	0.4
Steel wire	100,000	0.29
Kirschner wire	200,000	0.3
Anchor Suture	200,000 200	0.3 0.3

### Finite element analysis

**Fig. 2:**
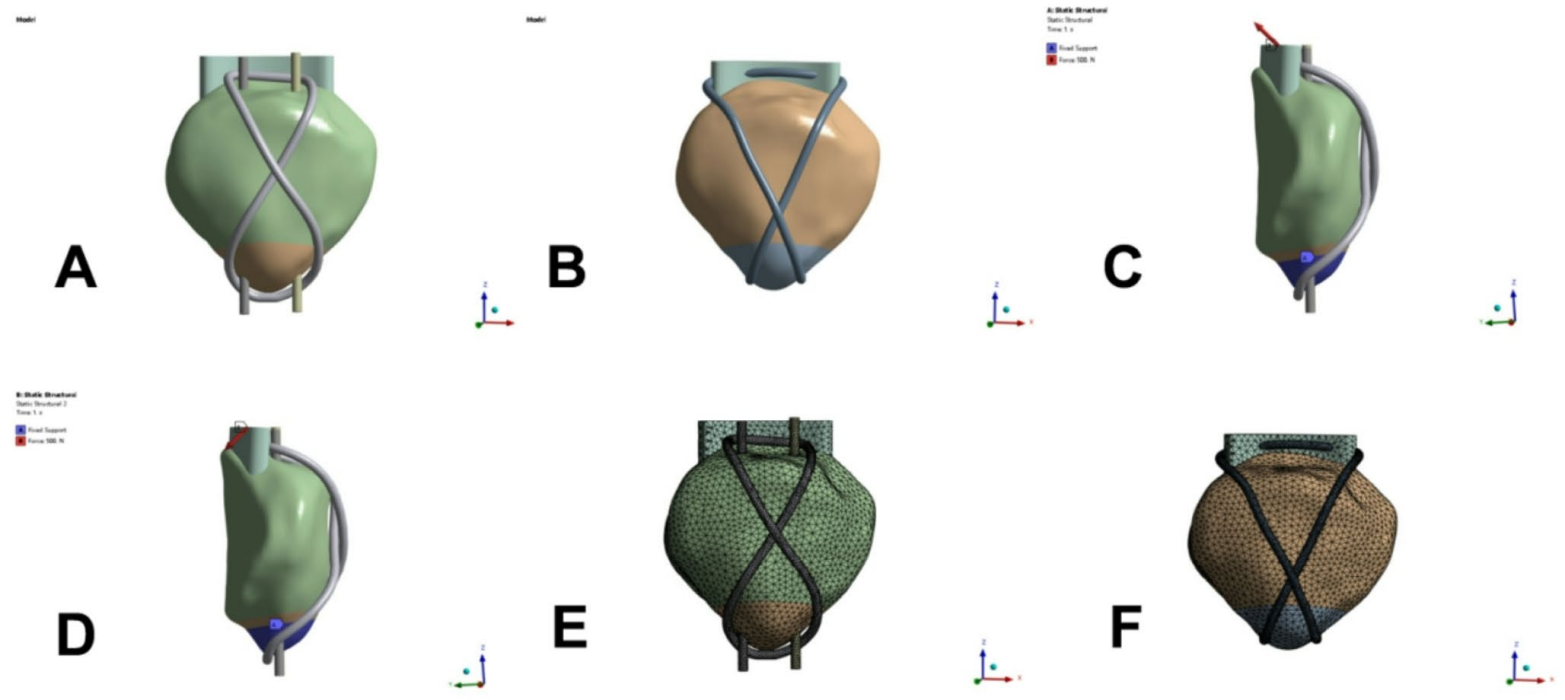
Schematic diagram of the mesh model and patellar load in finite element. (**A**, **E**) The mesh model of the TBW group, (**B**, **F**) The mesh model of the SA group, (**C**, **D**) The lower pole of the patella is restrained and the upper pole is loaded. The arrow indicates the direction of stretching. The blue circles represent support for the patella from the medial and lateral femoral condyle

According to the structural and biomechanical characteristics of the patella in this analysis, during normal knee joint movement, the patella mainly bears three forces, namely the upper pole quadriceps tendon, the lower pole patellar ligament tension, and the pressure between the patellofemoral articular surface [5]. In our analysis, the model was set up with non-weight-bearing knee extension at 45° and 135° of joint flexion. We constrained the inferior pole of the patella and applied a load on its superior pole to exert a single force of 500 N (Fig. 2C,D). For the convenience of study, it is assumed that all materials involved are isotropic and plastic deformation is not considered. Finally, the stress and displacement distribution of each model patella and internal fixation under 500N load was analyzed, and finally the cloud diagrams of equivalent force and displacement distribution were inserted to obtain the result diagrams of different fixation methods.

For the purpose of this comparative study, all materials were assumed to be isotropic and linearly elastic, with perfect bonding at all interfaces (e.g., between bone and implants, and between sutures and bone tunnels). This represents a simplified best-case scenario for both fixation techniques. While this approach does not fully capture the complex, non-linear behavior of living tissue, dynamic loading, or potential variables like suture slippage and soft tissue interaction in a clinical setting, it provides a standardized and controlled numerical environment. This allows for a direct and valid comparison of the inherent biomechanical properties of the two distinct fixation constructs by isolating them from confounding clinical variables.

### Surgical procedures

**Fig. 3:**
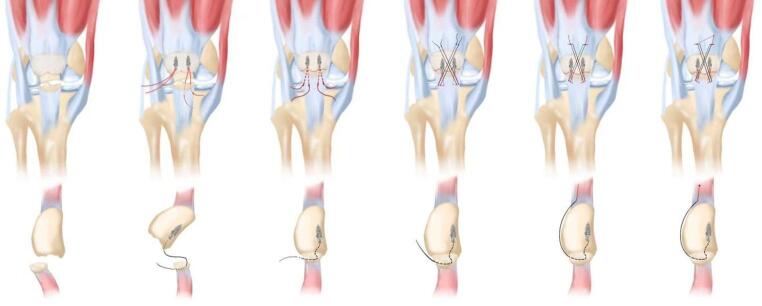
Schematic diagram of the operation. (**A**) Patella inferior fracture, (**B**) Insertion of wired anchors, (**C**) Alignment of fracture surfaces, (**D**) Formation of an "8" tension band for fixation, (**E**, **F**) Final reinforcement work

Patients in the SA group were operated on in the supine position, and a tourniquet was usually applied to the upper thigh. An anterior midline longitudinal incision is made from the superior pole to the inferior pole of the bone. The hematoma at the fracture end is then cleared, and two threaded anchors are screwed in at the proximal end of the fracture, close to the articular surface of the patella, with two sutures on each surface. Four rivet sutures were passed through the patellar tendon at even intervals from the dorsal side of the lower pole to the front, and were temporarily fixed after reduction using point-type reduction clamps. The fracture end was determined to be reduced under C-arm fluoroscopy. Then tie the rivet sutures in pairs to fix the fractured ends (if it is a comminuted fracture, you can use the "half purse" suture technique to make it into one piece with rivet sutures). Finally, suture one (two) bundles through the surface of the patella to the opposite side of the upper pole of the patella (the distance between the needle points is about 2–4 mm and tightly connected to the upper bone surface of the patella). After tying, sew the sutures to the upper and opposite sides of the patella. In the same way, sew the other two rivet sutures through the surface of the patella to the upper pole of the opposite side of the patella to form an "8" tension band. Then the knots are fixed, and finally the partially torn patellar ligament and patellar aponeurosis can be sutured and reinforced. Finally, the incisions were sutured layer by layer. All patients were followed up for at least 6 months postoperatively (Fig. 3). In the TBW group, standard Kirschner wire tension band technique was used for internal fixation.

## Results

### Finite element analysis

**Fig. 4:**
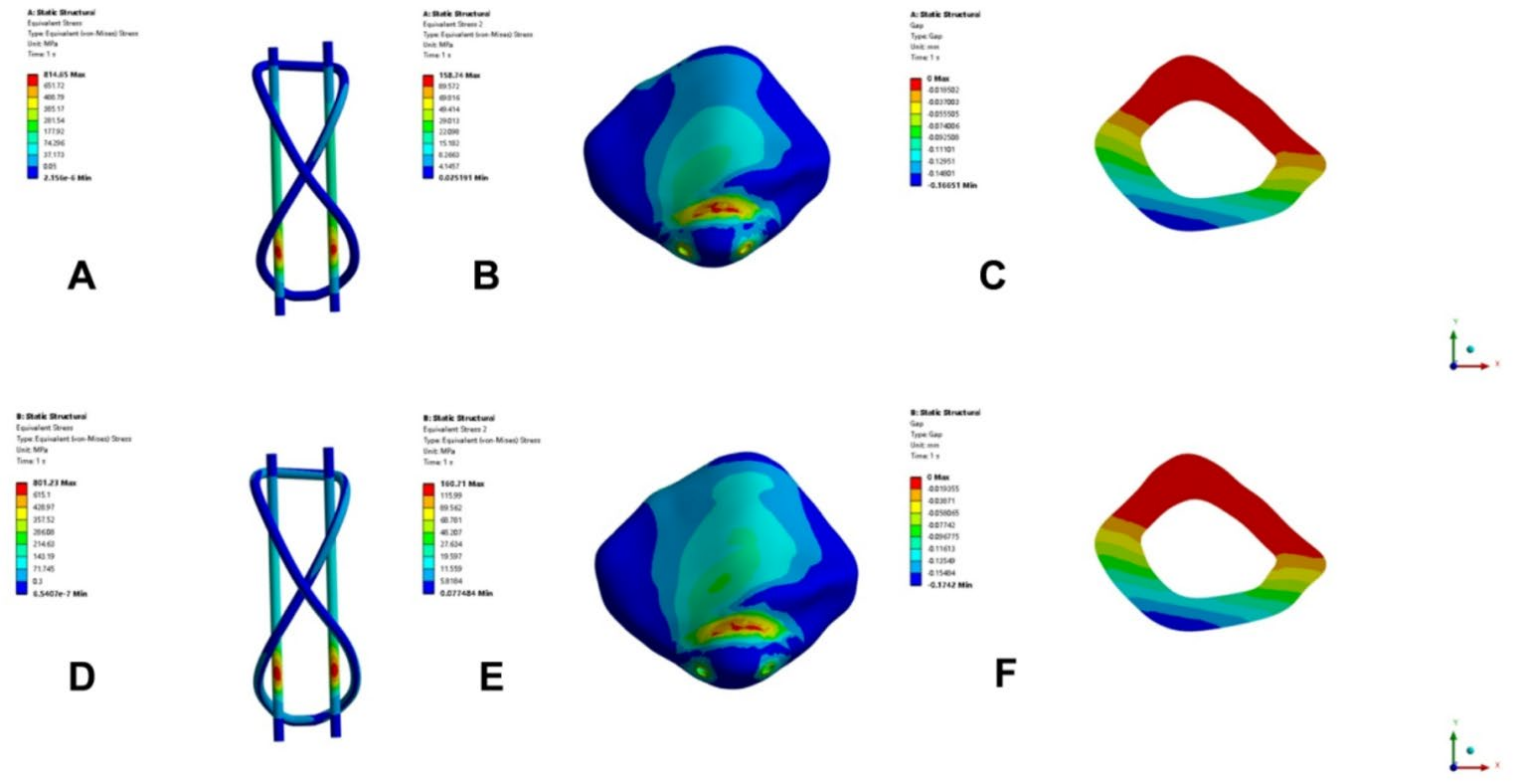
Displacement and stress cloud diagram of the TBW group under the action of 500N force at different angles. (**A**) Internal fixation stress cloud diagram at 45°, (**B**) Patella stress cloud diagram, (**C**) Fracture end gap cloud diagram, (**D**) Internal fixation stress cloud diagram at 135°, (**E**) Patella stress cloud diagram, (**F**) Fracture end gap cloud diagram

**Fig. 5:**
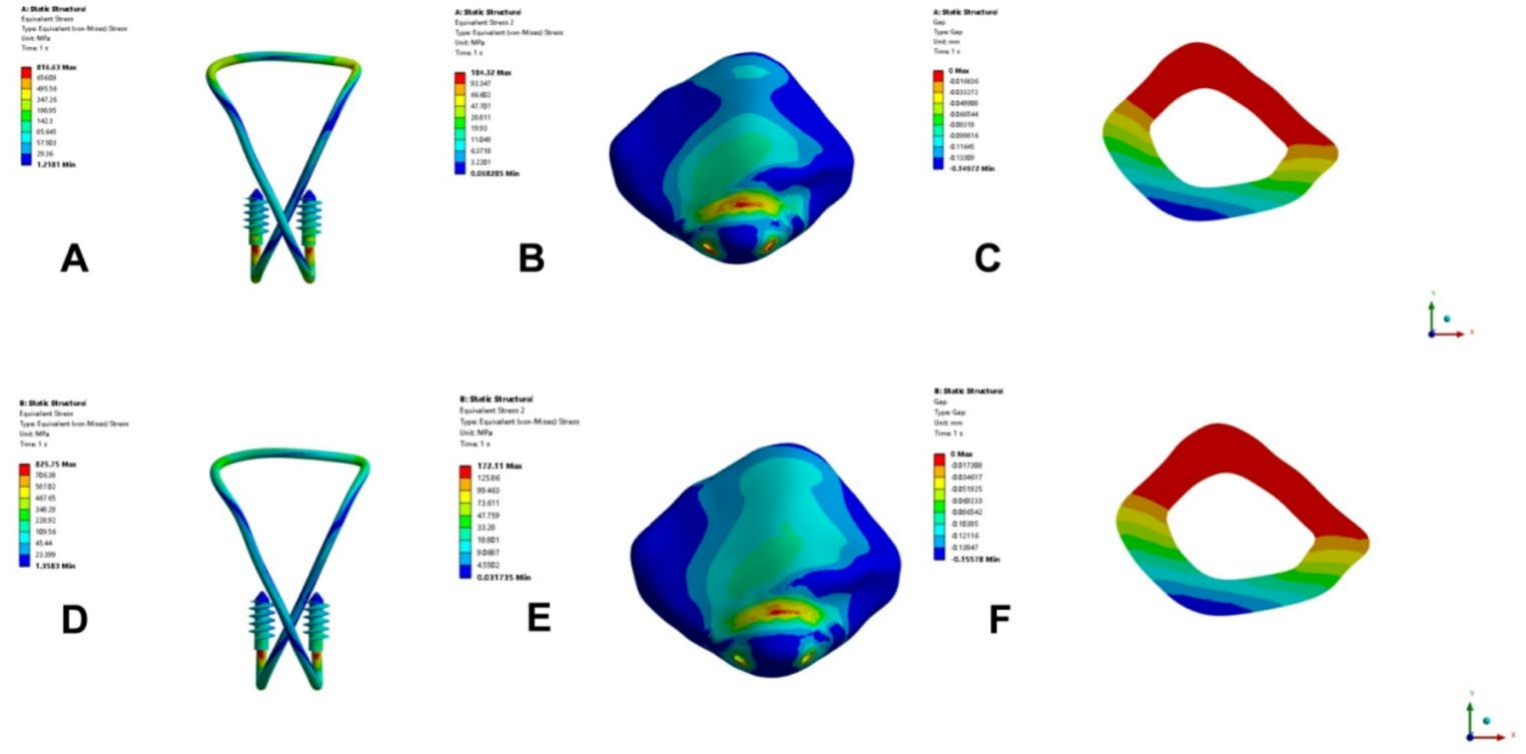
Displacement and stress cloud diagram of SA group under 500N force at different angles. (**A**) Internal fixation stress cloud diagram at 45°, (**B**) Patella stress cloud diagram, (**C**) Fracture end gap cloud diagram, (**D**) Internal fixation stress cloud diagram at 135°, (**E**) Patella stress cloud diagram, (**F**) Fracture end gap cloud diagram

After performing finite element analysis on the two models, we calculated and compared the stress and displacement results of different models under simulated knee flexion of 45° and 135° (Figs. 4, 5). The results are as follows: at 45° and 135°, the maximum fracture gap displacement under 500N load in the TBW group was 0.167 and 0.174 mm respectively (Fig. 4C, F); the maximum von Mises stress of the patella was 159 MPa and 161 MPa respectively (Fig. 4B, E); the maximum von Mises stress of internal fixation was 815 MPa and 801 MPa (Fig. 4A, D). The maximum fracture gap displacements in the SA group under 500N load were 0.150 and 0.156 mm respectively (Fig. 5C, F); the maximum von Mises stress of the patella was 184 MPa and 172 MPa respectively (Fig. 4B, E); the maximum von Mises stress of internal fixation was 817 MPa and 826 MPa (Fig. 4A, D).

### Clinical example

**Fig. 6:**
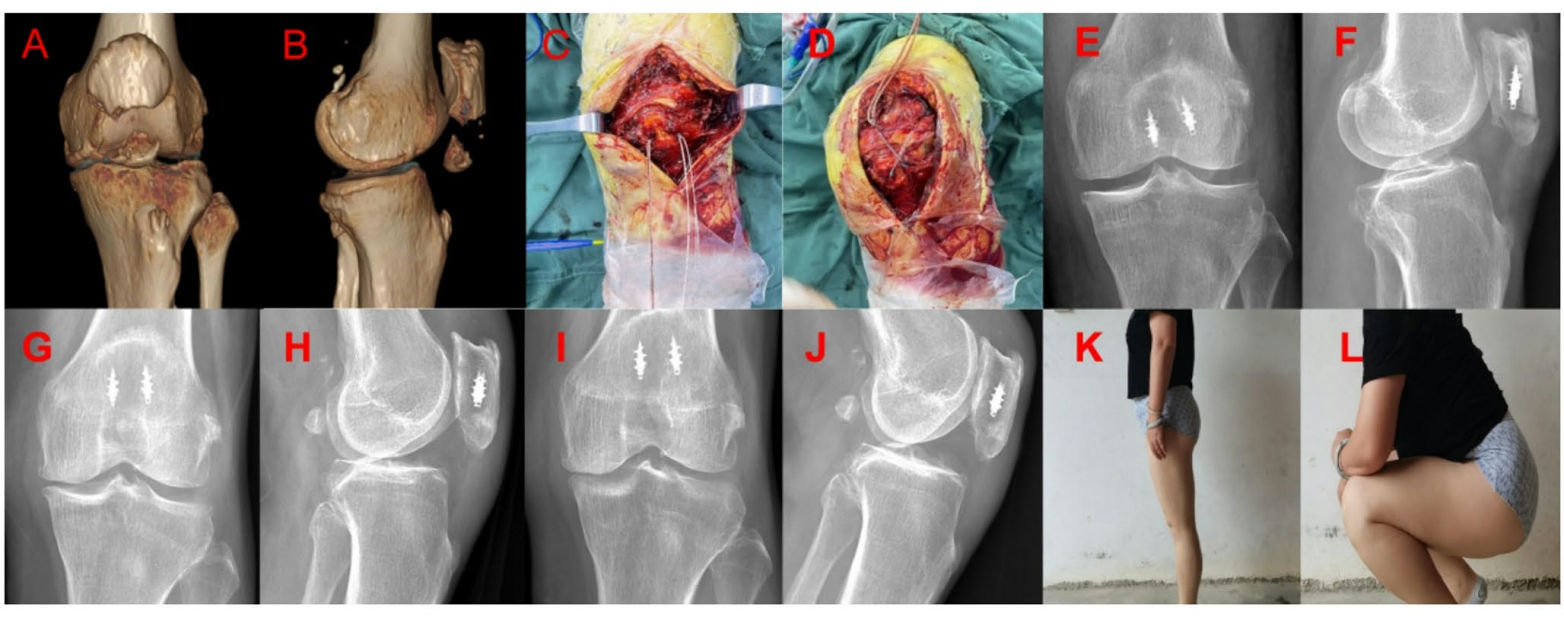
Clinical example. (**A**, **B**) Preoperative three-dimensional CT, (**C**, **D**) Intraoperative pictures, (**E**, **F**) Postoperative anteroposterior and lateral X-rays, (**G**, **H**) Postoperative anteroposterior and lateral X-rays 3 months, (**I**, **J**) Postoperative anteroposterior and lateral X-rays 6 months, (**K**, **L**) Postoperative functional photos

A representative patient treated with the SA technique completed 6 months of postoperative follow-up. Preoperative three-dimensional CT revealed a comminuted inferior pole fracture (Fig. 6A, B). Intraoperative photographs demonstrate the insertion of wired anchors and the formation of the "8" tension band (Fig. 6C, D). Postoperative anteroposterior and lateral radiographs showed satisfactory fracture reduction and implant position (Fig. 6E, F). Follow-up radiographs at 3 months (Fig. 6G, H) and 6 months (Fig. 6I, J) confirmed complete fracture healing with no loss of reduction or implant failure. At the final follow-up, the patient was able to perform normal knee bending and kneeling movements without pain or functional limitation (Fig. 6K, L).

## Discussion

Treatment of inferior pole fractures of the patella is challenging. Because the fracture fragments are often comminuted, the distal bone fragment is small, and the blood supply is relatively poor, conservative treatment is often ineffective, and surgical treatment has become the preferred option [12]. However, this site has the characteristics of high tensile stress concentration, strong traction force of the patellar tendon, and difficulty in effectively holding small bone fragments, making it difficult to achieve and maintain stable internal fixation [13].

Resection of the inferior pole of the patella will destroy the native biomechanical efficiency and can lead to adverse consequences such as weakness in knee extension, and its clinical application has gradually decreased [14]. TBW technology aims to convert anterior tension into pressure on the fracture surface, but its stress is concentrated at the K-wire-wire junction, and the problem of reduced pullout resistance is particularly prominent in patients with osteoporosis [15]. In addition, wire fixation may cause local stress concentration, increase the risk of cutting cancellous bone (especially in patients with osteoporosis), and has the potential to over-stimulate soft tissues, damage tendons, and cause fixation failure [16]. And a second-stage surgery is required to remove the implant. To overcome these limitations, a variety of improved techniques based on the tension band principle have been proposed. For example, Zhu et al. [17] used a steel plate structure to effectively offset longitudinal stress, and at the same time significantly reduced the risk of bone displacement through a tension band mechanism; however, the soft tissue irritation problem caused by the protrusion of the steel plate cannot be ignored. Yan et al. [18] used flexible steel cables to replace traditional steel wires, which reduces the risk of bone cutting through more uniform stress dispersion, which is more advantageous for patients with osteoporosis. However, the elastic modulus characteristics of the steel cables may sacrifice some immediate stability. Zhang et al. [19] used 2 parallel Kirschner wires to penetrate the patella longitudinally, combined with titanium cables to fix the lower pole bone in a figure-8 suspension manner, and decomposed the patellar tendon pulling force through vertical vectors to improve the anti-displacement ability. For patients with osteoporosis, a bone tunnel can be drilled in the lower pole of the patella, and the overlock suture method (Krackow method) can be used to penetrate the patellar tendon and then make a "8" cross, and fix the suture to the upper pole through the bone tunnel. This method can make the stress at the suture-bone interface more evenly distributed [20]. Although these improved technologies each have their own advantages and highlight the importance of the tension band principle, problems such as instrument protrusion or insufficient stability are still common [21].

Therefore, this study proposes a new surgical plan that may enhance the fixation of bone fragments through multi-directional suture tension, potentially reducing the risk of fragment displacement. During operation, care should be taken to ensure that the sutures run closely against the bone surface to avoid entrainment of soft tissue. Otherwise, the sutures may cut the soft tissue during postoperative functional exercises, resulting in loosening of the fixation or even separation of the fracture fragments. What is particularly critical is that this technology achieves the optimization of tensile stress at the fracture fragment interface through multi-directional and multi-angle suture tension. This mechanism effectively avoids the common drawbacks of traditional steel wire "point" fixation methods (excessive stress concentration on a single fixation point), thereby significantly reducing the risk of fixation failure due to stress concentration. For complex cases such as lower pole comminuted fractures, the "half purse" suture technique can be used to assist in achieving stable fixation of the fracture fragments. In addition, based on the finite element analysis results, the novel "8" cross-wound tension band technology (SA group) proposed in this study has the potential to be a viable alternative to the traditional steel wire tension band (TBW group). Under the 500N load, there was no significant difference in any mechanical index between the SA group and the TBW group in the two stages representing the early (45°) and larger range (135°) knee flexion functions. Specifically, the maximum fracture gap displacement (SA group: 0.150 mm & 0.156 mm; TBW group: 0.167 mm & 0.174 mm) and the maximum von Mises stress of internal fixation (SA group: 817 MPa & 826 MPa; TBW group: 815 MPa & 801 MPa) of the two models at different angles are highly similar. This result suggests that in the entire range of functional activities from moderate knee flexion to deep knee flexion, the fixation stability and built-in load of the new technology are comparable to those of traditional surgery, and there is no drastic change in performance as the angle increases. This shows that the mechanical environment provided by the new technology is equally safe and reliable throughout the critical activity range of postoperative rehabilitation, and will not increase the risk of fixation failure due to the increase in knee flexion angle. Although the patella stress of the SA group (184 MPa and 172 MPa) is slightly higher than that of the TBW group (159 MPa and 161 MPa), its value is still far below the yield limit of cortical bone and does not affect the overall safety of fixation.

The core advantage of the new technology is that while it provides mechanical properties comparable to those of traditional surgery, it also optimizes stress distribution through "8" cross-winding, and avoids metal implant irritation by virtue of its fully implanted design, saving patients from secondary surgery. Based on the strong support of the above finite element analysis, the core advantages of the new surgical plan proposed in this study have been quantitatively confirmed. In addition, this fully implantable fixation system eliminates the soft tissue irritation issues associated with metal implants and eliminates the need for secondary surgeries. In terms of postoperative rehabilitation, the stability and strength confirmed by finite element analysis provide confidence for early rehabilitation: it is recommended to gradually perform active knee flexion exercises under the guidance of a physician starting 1 week after surgery. In summary, this technology is not only innovative in clinical operation, but its superiority has been verified at the mechanical level through rigorous finite element analysis, showing significant comprehensive advantages in reducing the risk of complications and promoting early functional recovery.

The limitations of this study are mainly reflected in the following aspects. First, the patella model was reconstructed based on CT data of a single healthy young male volunteer without knee joint trauma or disease, lacked individual heterogeneity of human anatomy (such as differences in patella bone density, morphological structure, and bone quality), and could not fully represent the diverse clinical characteristics of patellar inferior pole fractures (such as patients with varying degrees of comminution, fracture displacement, and osteoporosis). Secondly, to simplify FEA calculations, this study assumes that all biological and implant materials are isotropic and linear elastic, and plastic deformation is not considered. In fact, cortical bone and cancellous bone are typical anisotropic materials, and the mechanical interaction at the bone-implant interface in the in vivo environment is more complex, which may lead to certain deviations between the simulation results and the actual physiological state. Third, the load condition in this study was set as a static single load of 500 N at two fixed knee flexion angles (45° and 135°), which failed to simulate the dynamic, multidirectional, variable-size loads experienced by the knee joint in actual daily activities (such as walking, climbing stairs, squatting, etc.). In the future, more sophisticated modeling strategies incorporating nonlinear material properties, dynamic loading, and soft tissue interactions are needed to improve the fidelity of biomechanical simulations. These findings should ideally be confirmed by larger, multicenter randomized controlled trials to fully assess the clinical effectiveness and long-term safety of this combined fixation approach.

## Conclusion

Based on finite element analysis and biomechanical evaluation, the proposed novel technique shows potential as a viable fixation option for inferior pole patellar fractures. From a biomechanical perspective, this composite fixation method has comparable stability and fixation strength to traditional tension band wires when the knee joint is flexed at 45° and 135°, providing a solid theoretical basis for its clinical application. However, it should be noted that the finite element model in this study involves inherent simplifications and therefore the simulation results should be interpreted with caution. Future research should focus on developing a more physiologically representative finite element model, combined with in vitro biomechanical testing and large-sample prospective clinical trials, to more comprehensively evaluate the clinical efficacy and safety of this technology and provide a higher level of evidence for its wider clinical application.

## Data Availability

The datasets used and/or analysed during the current study are available from the corresponding author on reasonable request.
